# How action selection can be embodied: intracranial gamma band recording shows response competition during the Eriksen flankers test

**DOI:** 10.3389/fnhum.2014.00668

**Published:** 2014-08-26

**Authors:** Fausto Caruana, Sebo Uithol, Gaetano Cantalupo, Ivana Sartori, Giorgio Lo Russo, Pietro Avanzini

**Affiliations:** ^1^Brain Center for Social and Motor Cognition – Istituto Italiano di TecnologiaParma, Italy; ^2^Department of Neuroscience, University of ParmaParma, Italy; ^3^Department of Life and Reproduction Sciences, University of VeronaVerona, Italy; ^4^“Claudio Munari” Center for Epilepsy Surgery, Ospedale Niguarda-Ca’ GrandaMilan, Italy; ^5^Department of Biomedical, Metabolism, and Neural Science, NOCSAE Hospital, University of Modena and Reggio EmiliaModena, Italy

**Keywords:** embodiment, action selection, intracerebral recording, human PMC

## Abstract

Recent findings in monkeys suggest that action selection is based on a competition between various action options that are automatically planned by the motor system. Here we discuss data from intracranial EEG recordings in human premotor cortex (PMC) during a bimanual version of the Eriksen flankers test that suggest that the same principles apply to human action decisions. Recording sites in the dorsal PMC show an early but undifferentiated activation, a delayed response that depends on the experimental conditions and, finally, a movement related activation during action execution. Additionally, we found that the medial part of the PMC show a significant increase in response for ipsilateral trials, suggesting a role in inhibiting the wrong response. The ventral PMC seems to be involved in action execution, rather than action selection. Together these findings suggest that the human PMC is part of a network that specifies, selects, and executes actions.

## INTRODUCTION

In the cognitivist’s framework, action decisions stem from a conveniently arranged system. The central cognitive module evaluates the input from the visual areas, selects a specific action, and subsequently informs the premotor cortex (PMC) for action planning and execution (Miller et al., 1960; see Fodor, 1983). There is abundant evidence that this now infamous “sandwich model” of cognition is empirically untenable (Uithol et al., 2012, 2014; Engel et al., 2013; Schurger and Uithol, in press), and very few scientists today would explicitly endorse such a view on cognition.

The sandwich model is by and large replaced with embodied approached to cognition. Within these approaches cognition is considered to be something that is not detached from perception and action, but something that emerges from the tight connection between these processes. However, these embodied approaches face a new problem with respect to action control: how can embodied processes result in a cognitive decision to perform a certain action? There is no central decision center that is planning the action, but at the same time it is highly unlikely that decisions are purely context driven sensorimotor links. How can action decisions be embodied and cognitive at the same time?

Electrophysiological recordings in monkeys gave rise to an interesting model that could explain how decisions can come about in an embodied cognitive system (Cisek, 2007; Cisek and Kalaska, 2010). In this “affordance competition hypothesis” the observation of a stimulus suggesting multiple competing actions elicits the parallel planning of all possible actions. Importantly, these action options are concrete action plans, specified for the appropriate context across fronto-parietal circuits. The options compete for further processing and are biased by input from prefrontal cortical regions and the basal ganglia. A decision in this framework is the prevalence of one option – through biasing influence of context features and internal processes – and the suppression of the others (see also Crammond and Kalaska, 1994; Gail et al., 2009). Recent evidence suggests that similar processes underlie human decision processes. Michelet et al. (2010) showed that the corticospinal excitability during simple decisions (i.e., a Eriksen flankers test) reflects a competition between action options. While these important findings suggest a similar mechanism at work in human action selection, they do not provide details on the source and dynamics of these selection processes.

Here we want to discuss the first evidence of a competition between action options using intracranial recordings in humans, and discuss the temporal dynamics underlying this process. We used a bimanual version of the classic Eriksen flankers test (Eriksen and Eriksen, 1974). This classic test consists in the presentation of a directional arrow, flanked by congruent, or incongruent, similar directional arrows, or by neutral stimuli. Subjects are asked to respond as fast as possible in a way that is congruent to the direction indicated by the central arrow, and independently by the flanked distractors. Behavioral studies showed that the response is delayed when the central cue is flanked by incongruent directional stimuli, and the increase in reaction time is considered to reflect the interference between competing responses. During this test we recorded high gamma-band activity (50–150 Hz) from six patients with frontal implantations involving the PMC. We found (1) an early but non-specific response to an observed stimulus (100 ms after stimulus onset) of PMC neurons, (2) a delayed activation, modulated by the experimental condition, and (3) a modulation depending on the subsequent action. Together, these findings suggest an action selection process in the motor system as suggested in the affordance competition hypothesis.

## MATERIALS AND METHODS

### PARTICIPANTS

The experiment was performed on six patients (gender: *F* = 3; *M* = 3; age: 23 ± 9; implantation side: right = 1; left = 5) suffering from drug-resistant focal epilepsy and stereotactically implanted with intracerebral electrodes as part of their pre-surgical evaluation, at the “Claudio Munari” Center for Epilepsy Surgery, Ospedale Niguarda-Ca’ Granda, Milan, Italy. Implantation sites were selected on purely clinical grounds, on the basis of seizure semiology, scalp-EEG, and neuroimaging studies, and with no reference to the present experimental protocol. Patients were fully informed of the electrode implantation and stereo-EEG recordings, and, according to the Declaration of Helsinki (*BMJ* 1991; 302: 1194) gave written informed consent to participate in the study. Experimental procedures were approved by the Ethical-Scientific Committee of the Ospedale Niguarda-Ca’ Granda. We selected patients whose precentral region was not affected by epileptic activity. No seizures were recorded during the 24 h prior to the experiment. No alteration in the sleep/wake cycle was observed, and no additional pharmacological treatment was applied before the experiment. Patients did not show any motor or cognitive deficits during the examination; they fully understood the instructions and easily performed the experimental task.

### ELECTRODE IMPLANTATION

For each patient, up to fifteen depth electrodes were implanted in different regions of the brain including the PMC. To reach the clinically relevant targets, the stereotactic coordinates of each electrode were calculated preoperatively based on the individual’s cerebral MRI. Each electrode had a diameter of 0.8 mm and comprised 10–15 2 mm long contacts, spaced 1.5 mm apart (DIXI^®^, Besançon, France). Cerebral structures explored by each electrode contact were determined by coregistration of pre-implantation volumetric brain MRI with post-implantation volumetric brain CT, and visualized by a software package for visualization and image analysis (3DSlicer^®^).

### PROCEDURE

**FIGURE 1:**
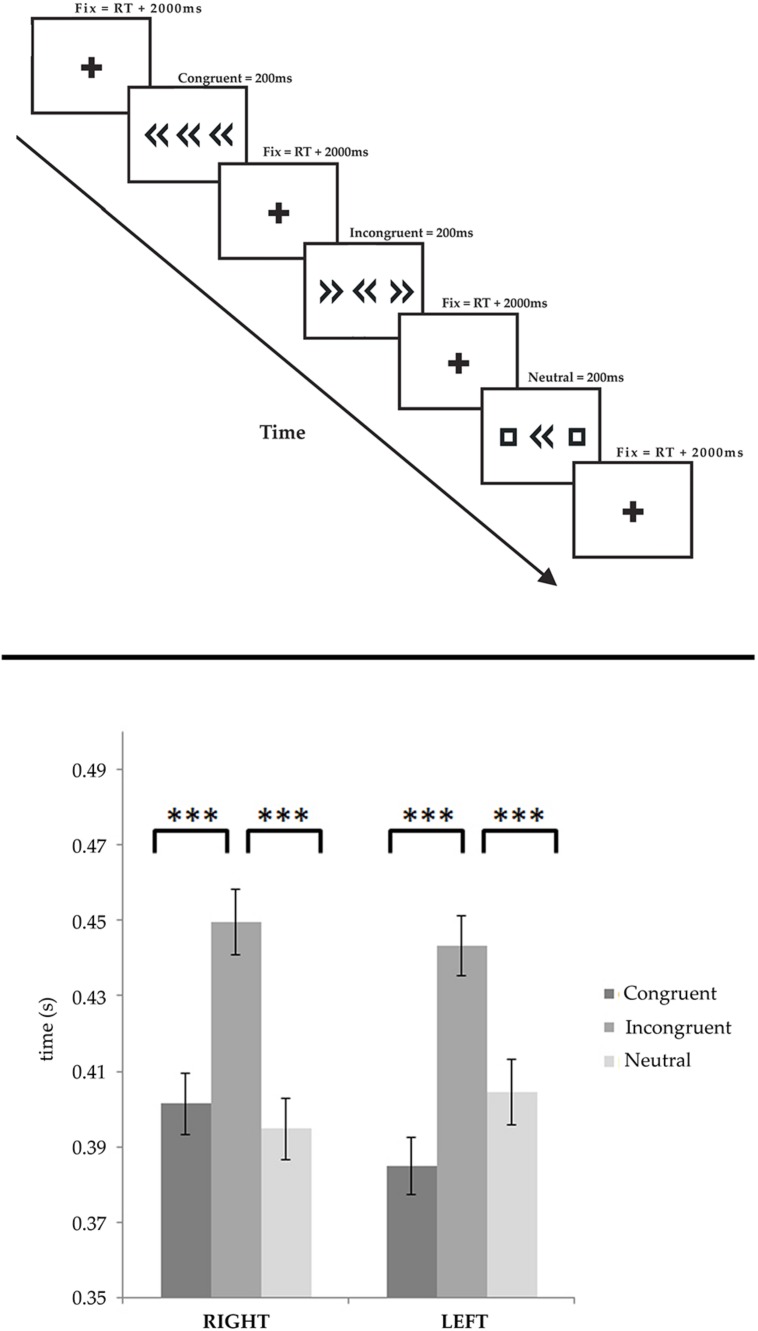
**(Upper panel)** Experimental paradigm. The stimuli consisted in a horizontal array of three symbols consisting in a central arrow (target), flanked by identical arrows in the same (congruent) or opposite (incongruent) direction, or by squares composed by the same graphical elements of the target arrows, but without any directional information (neutral condition). Each stimulus was presented for 200 ms and followed by a fixation cross lasting the response time plus two additional seconds. **(Lower panel)** Behavioral results are shown for both the left and right responses, separately. In both cases the incongruent trials elicited significantly delayed responses, as compared to the congruent and neutral trials. No condition showed any significant effect of side. ***p < 0.0001.

Recordings were obtained in a dimly light quiet room. The patient sat approximately 100 cm away from the laptop display where the stimuli were presented. The stimuli consisted in a horizontal array of three symbols consisting in a central arrow (target), directed toward right or left, flanked by identical arrows in the same (congruent) or opposite (incongruent) direction, or by squares composed by the same graphical elements of the target arrows, but without any directional information (neutral condition). Consequently six conditions were presented: right congruent (RC), right incongruent (RI), right neutral (RN), left congruent (LC), left incongruent (LI), and left neutral (LN). Each stimulus was presented in random order for 200 ms after a fixation cross of 2000 ms (see **Figure 1**, upper panel). Each condition was presented 60 times. During the task the hands of the patients leaned on the keyboard, with the index fingers on the two buttons located at the top right and top left side of the keyboard (the “Esc” and “Del” button). The patient was asked to press the correct button (the button congruently to the central target) as fast as possible using the corresponding hand. A short practice session (<2 min) took place before the start.

### STEREO-EEG RECORDING AND ANALYSIS

During the experiment continuous stereo EEG (sEEG) was recorded with a 1000 Hz sampling rate by means of a 192 channel-EEG device (EEG-1200 Neurofax, Nihon Kohden^®^). Each channel was referred to a contact in the white matter far from the recording sites, in which low and high frequency electrical stimulations did not produce any subjective or objective manifestation (neutral reference). At the end of each experimental session, sEEG data were exported and the activity of each contact located in the precentral region (*n* = 46 recording sites) was selected. A visual inspection was carried out by clinicians in order to ensure the absence of any pathological interictal activity. Trials showing artifacts were removed. A band-pass filter (0.015–500 Hz) was applied to avoid any aliasing effect. Each trial was epoched with a (-500, 1000) ms time window, with respect to the image onset. Activity in the broad gamma band (50–150 Hz) was analyzed in the time-frequency (TF) domain by convolution with complex Morlet’s wavelet. The broad gamma band is a typical feature of intracranial-EEG (iEEG) recordings in epileptic patients, and spatially and functionally more specific than the power modulation in other bands, or in the intracranial ERP (iERP; Vidal et al., 2010; Caruana et al., 2014a,b). Furthermore it is currently associated to neuronal population spiking activity (Manning et al., 2009; Ray and Maunsell, 2011), and to the BOLD signal as well (Logothetis et al., 2001; Lachaux et al., 2007). In line with our previous intracranial studies (Caruana et al., 2014a,b) gamma power was estimated for 10 adjacent non-overlapping frequency bands, each 10 Hz wide, and a divisive baseline correction was applied versus the prestimulus interval (-500/0). The entire analysis pipeline was performed by integrating EEGLAB^®^ functions with home-made Matlab^®^ code.

### STATISTICAL ANALYSIS

The analysis was performed on all the contacts located in BA6, according to their MNI coordinates. Forty-six contacts, out of 574 implanted ones, were located in different sections of the precentral gyrus; more specifically seven were located in the vPMC, 22 were located in the dPMC and 17 were located in the medial parts of Brodmann Area 6 (mBA6; see **Table 1** for the localization of the entrance points). To evaluate the presence of early visual responses in the precentral region we preliminarily calculated with a one-sample *t*-test the significance of gamma band power values versus a zero-mean distribution, using time bins of 20 ms each, in a (-100, 100) ms time window. The analysis was applied to the six different conditions, separately. This pre-scanning was aimed to evaluate whether BA6 sites present any early response to the task regardless the between conditions differences. Given the lack of conventional analysis in sEEG we adopted this procedure from electrophysiological studies on single unit recordings in the monkeys, as sEEG recordings resemble such technique concerning the inhomogeneous spatial sampling and the spatial selectivity of the activity. A similar approach on sEEG data was used by our group in the past (Caruana et al., 2014b). All contacts showing a significant gamma modulation in at least two consecutive time bins in any of the conditions were considered for more detailed analyses. More specifically, to evaluate whether the early visual response was modulated by the incongruent, congruent or neutral conditions, a repeated measures ANOVA was applied to each significant contact, considering CONDITION (congruent, incongruent, and neutral) and TIME [10 adjacent 20 ms time bins (-100, 100)] as factors, for both the contralateral and the ipsilateral responses. In order to account for the multiple comparisons issue, the p-threshold used to consider an ANOVA effect (either main effect or interaction) as significant was Bonferroni corrected dividing the standard 5% value for the number of carried out independent analyses, i.e., the amount of significant leads. For each significant interaction, *post hoc* analysis was conducted by means of a paired *t*-test.

**Table 1:** Electrode entrance points.

Entrance points			
Patient	Electrode	MNI	Hemisphere
P1	F	-52.0, -0.8, 46.9	L
P1	K	-19.9, 19.5, 59.0	L
P1	M	-31.0, -15.7, 69.2	L
P2	J	-16.7, 21.1, 58.4	L
P2	L	-16.7, 21.1, 58.4	L
P3	H	-58.9, 3.4, 32.4	L
P3	M	-42.7, -8.9, 59.4	L
P4	M	48.1, -5.6, 52.4	R
P5	M	-57.8, -1.3, 36.5	L
P6	N	-49.9, -1.1, 48.5	L

*The MNI coordinates of the entrance points and the side of implantation of each electrode are shown.*

A second analysis was aimed to evaluate the presence of a delayed response, during the action selection process. First we calculated with a one-sample *t*-test the significance of gamma band power values versus a zero-mean distribution, in a (-500, 1000) ms time window. As this time window was much larger, we decided to increase the single time bin size to avoid an overdetailing of a spreaded-over-time effect, using time bins of 100 ms each. The analysis was applied to the three conditions (congruent, incongruent, and neutral) requiring an ipsilateral response. Only the correct trials were analyzed. The same analysis was not applied to the trials requiring a contralateral response to avoid the risk of false positives due to the delayed motor responses in the incongruent trials. All contacts showing a significant gamma modulation in at least one time bin in one of the three conditions were considered task-related and considered for a more detailed analysis. More specifically, to evaluate whether the late response was modulated by the incongruent, congruent or neutral conditions, a repeated measures ANOVA was applied to each significant contact, considering CONDITION (congruent, incongruent, and neutral) and TIME [30 adjacent 50 ms time bins (-500, 1000)] as factors. For each significant interaction, *post hoc* analyses were performed by means of a paired *t*-test.

Finally, to evaluate whether the contacts showing a response during the action selection process were also involved in the action execution, we statistically compared their gamma band modulation during trials requiring a responses with the ipsilateral hand to the trials requiring a response with the contralateral hand, grouping together the three conditions. A repeated measures ANOVA was applied considering HAND (contralateral, ipsilateral) and TIME [30 adjacent 50 ms time bins (-500, 1000)] as factors. ANOVA *p*-values were not corrected for the number of explored leads because, differently from scalp and non-invasive recordings, sEEG records local activity that is rather independent among different leads. In contrast, for each lead exhibiting a significant TIME ^∗^CONDITION interaction, we run a *post hoc* analysis by means of paired *t*-test.

## RESULTS

### BEHAVIORAL STUDY

We performed a repeated measures ANOVA on RTs measured during iEEG recording in both rightward and leftward responses, considering the SIDE (rightward vs. leftward) and the CONDITION (congruent, incongruent, and neutral) as factors. Wrong responses were discarded from the analysis. The analysis showed a clear influence of the factor condition on RT [*F*(2,442) = 52.232, *p* < 0.0001], but we did not find any significant effect of side [*F*(1,221) = 0.02318, *p* = 0.87913]. *Post hoc* comparisons shown that RTs were significantly longer in the incongruent condition (449.6 ± 8 ms and 443.2 ± 8 ms, for rightward and leftward, respectively) than in the congruent (401.5 ± 8 ms and 384.9 ± 8 ms) and neutral conditions (394.7 ± 8 ms and 404.4 ± 9 ms; *p* < 0.0001). Furthermore, no significant differences were found between the rightward and leftward responses, within each experimental condition (see **Figure 1**, lower panel).

### SHORT LATENCY RESPONSES IN dPMC

**FIGURE 2:**
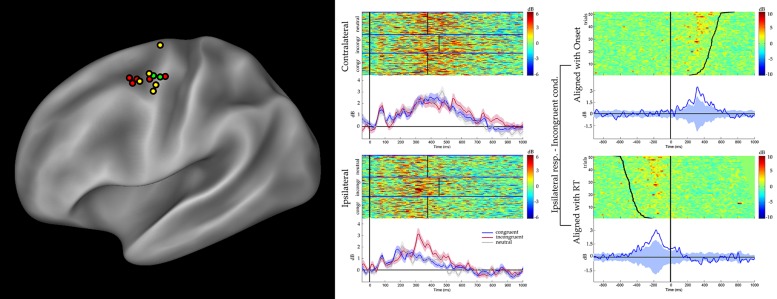
**(Left panel)** Sites showing significant unspecific, short- latency activations according to the gamma band reactivity within 100 ms after stimulus onset, are shown in yellow. Choice-related sites, showing significant activation during the incongruent trials, are shown in red. Furthermore, sites showing both effect are shown in green. Results are plotted on a template (Caret^®^) according to their MNI coordinates. **(Central panel)** Results from a representative d premotor cortex (PMC) site are shown (P3, M’12). Figure shows the gamma modulation during the congruent, incongruent, and neutral trials requiring a response with the hand contralateral (top) and ipsilateral (bottom) to the implanted hemisphere. Data are aligned with the stimulus onset. The average reaction time for the three conditions is shown at the single trial level (black vertical lines). The short-latency activations is clear in both contralateral- and ipsilateral- response trials, in the three conditions. In the contralateral-response trials the activity is prolonged after the response, while in the ipsilateral-response trials is suppressed before the response. The greater activity during the incongruent conditions is visible in the ipsilateral-response trials. **(Right panel**) The same dataset, showing a representative dPMC site (P3, M’12) during the incongruent condition requiring a response with the ipsilateral hand, aligned to both the onset of the stimulus (upper part) and the onset of the response (lower panel). For each selected alignment, the other event is indicated by a curved line.

A preliminary analysis was aimed to evaluate a significant gamma modulation within the early 100 ms per each contact and condition, separately. This analysis showed that only seven contacts out of 46 had a significantly stronger gamma modulation in at least two consecutive time bins in at least one of the six conditions. All of them were located in the dPMC (see **Figure 2**, left panel, and **Table 2**), while the vPMC and the mBA6 did not show any early activation. The repeated measures ANOVA performed to these contacts in both the contralateral and the ipsilateral trials showed a significant effect of TIME for all the investigated contacts (*p* < 0.05 Bonferroni corrected). In contrast, the effect of CONDITION, as well as the CONDITION × TIME interaction, were not significant in any of the investigated contacts.

**Table 2:** Results.

**Short latency responses**						**Movement related responses**				
P1	F’10	-32.7, -5.1, 44.6	L	dPMC		P1	M’1	-5.0, -13.8, 54.9	L	mBA6
P1	M’6	-18.1, -18.2, 69.1	L	mBA6		P1	M’2	-4.1, -16.4, 56.6	L	mBA6
P3	M’11	-35.9, -7.8, 54.6	L	dPMC		P1	M’3	-5.8, -20.8, 57.5	L	mBA6
P3	M’12	-42.3, -8.1, 59.3	L	dPMC		P1	M’4	-6.7, -22.0, 55.2	L	mBA6
P4	M8	36.9, -7.3, 53.8	R	dPMC		P1	M’5	-15.9, -18.9, 71.0	L	mBA6
P4	M9	42.6, -6.6, 53.8	R	dPMC		P1	M’6	-17.1, -18.3, 68.6	L	mBA6
P4	M10	44.8, -7.3, 51.2	R	dPMC		P1	M’8	-26.1, -16.0, 68.5	L	mBA6
						P3	M’6	-25.7, -1.4, 47.2	L	dPMC
						P3	M’7	-28.1, -1.2, 48.0	L	dPMC
**Choice related responses**						P3	M’9	-31.7, -4.7, 45.2	L	dPMC
P3	M’6	-26.1, -1.7, 47.2	L	dPMC		P3	M’10	-34.1, -8.3, 50.7	L	dPMC
P3	M’7	-28.8, -1.5, 48.0	L	dPMC		P3	M’11	-35.3, -8.3, 54.5	L	dPMC
P3	M’9	-31.7, -4.7, 45.2	L	dPMC		P3	M’12	-41.2, -7.1, 58.5	L	dPMC
P3	M’10	-35.2, -7.9, 51.6	L	dPMC		P3	M’13	-42.7, -8.9, 59.4	L	dPMC
P3	M’11	-35.9, -7.8, 54.6	L	dPMC		P4	M11	46.3, -4.1, 51.8	R	dPMC
P3	M’12	-42.3, -8.1, 59.3	L	dPMC		P5	M’13	-53.5, 0.5, 35.9	L	vPMC
P3	M’13	-42.7, -8.9, 59.4	L	dPMC		P5	M’14	-56.6, -0.6, 37.3	L	vPMC

*The MNI coordinates of the significant sites, the side of implantation of each electrode, and its anatomical region, are shown for the three main statistical analyses.*

### CHOICE-RELATED RESPONSES IN dPMC

In the behavioral Eriksen flankers task the incongruent condition produces a delayed response, compared to the congruent and the neutral conditions, and the increase in reaction times is considered to reflects the interference between competing responses. Accordingly, the second set of analyses was aimed to evaluate how action selection processes are accommodated in the human motor system, and more specifically to assess whether the decision to respond (or to inhibit a response) affected the gamma band in the recorded contacts. As a preliminary analysis we selected the task-related contacts by means of a *t*-test applied to the congruent, incongruent, and neutral conditions requiring a response with the hand ipsilateral to the implanted hemisphere. Trials with errors or with a delayed response (RT > 1 s) were discarded. Results showed that 26 out of 46 contacts showed a significant response. Fourteen contacts were located in the dPMC, 10 in the mBA6 and 2 in the vPMC. The repeated measures ANOVA performed on these contacts showed a significant effect of TIME (*p* < 0.001 Bonferroni corrected) in all the investigated contacts, and a significant CONDITION^∗^TIME interaction in 7 out of 26 contacts (*p* < 0.05 Bonferroni corrected; see **Figure 2** and **Table 2**). *Post hoc* comparison showed a significant increase of gamma modulation in the incongruent vs. the other two conditions. All the significant contacts were located in the left dPMC.

### MOVEMENT-RELATED RESPONSES

Finally we evaluated whether the contacts that measured activity during action selection also measured activity during action execution. To this purpose, we grouped together all the responses according to whether they required a contralateral or an ipsilateral response, with respect to the recording site. The ANOVA showed a significant CONDITION^∗^TIME (*p* < 0.05 Bonferroni corrected) interaction in 17 out of 46 contacts (eight in dPMC, two in vPMC, and seven in mBA6; see **Table 2**). *Post hoc* comparison showed that dPMC and vPMC sites showed a significantly prolonged activity during the contralateral trials, starting around 400 ms (i.e., while the subject were giving their manual response), thus suggesting a role of these regions in movement execution.

**FIGURE 3:**
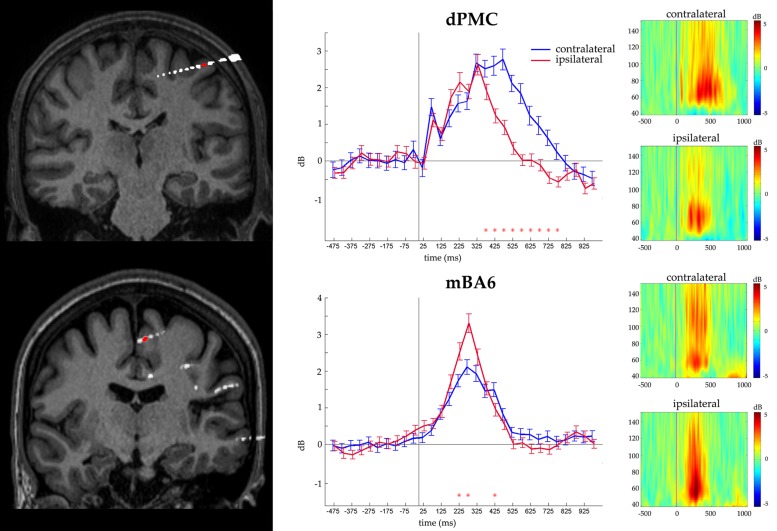
**Representative recording from a dPMC (top) and a mBA6 (bottom) sites. (Left panel)** The exact localization of the recording site is shown by the coregistration of the individual MRI with post- implantation CT. **(Central panel)** Gamma activity recorded from the contacts in red. Images show the statistical comparison between trials from the three condition pooled together, and requiring a contralateral vs. ipsilateral response in a -500, 1000 time window, in 30 adjacent time bins of 50 ms. Responses are aligned on the stimulus onset. Red asterisks indicate the significant *post hoc* comparisons. **(Right panel)** Time-frequency plots of the gamma activity (50–150 Hz) showing the frequency profile of the same electrodes, during contralateral and ipsilateral responses, in a -500, 1000 time window.

In contrast mBA6 sites showed a significantly stronger activity during trials requiring a response with the ipsilateral hand, and much earlier as compared to the other regions, that is, from 150 to 300 ms, thus suggesting an involvement of mBA6 in the inhibition of the unrequested movement (see **Figure 3**).

## DISCUSSION

In the present study we intracranially recorded from the dPMC, vPMC, and mBA6 during a bimanual version of the classic Eriksen flankers test (Eriksen and Eriksen, 1974). Only the dPMC appeared to play a specific role in action selection and showed a complex temporal pattern of response in the time window between the onset of the stimulus and the action execution. This complex response included (1) an early and unspecific activation, with a short latency within 100 ms from the onset of the stimulus; (2) a second activation, showing a modulation depending on the experimental condition, possibly reflecting an action selection process, and (3) a movement-related prolonged modulation when the response was given with the hand contralateral to the recording site.

The very first question concerns the nature of the first early activation of the dPMC. At a first glance, the three distinct neuronal events appear to be a human counterpart of the signal-related, set-related and movement-related activity described in the monkey dPMC neurons (Wise, 1985; Boussaoud and Wise, 1993). However, in contrast to the classic signal-related neurons, firing at the appearance of the instruction stimulus and differently modulated according to the instructed movement (Kalaska et al., 1997; Wise et al., 1997), the early modulation we have found is unspecific to the type of stimulus. Furthermore, its latency is too short to be a human counterpart of a signal-related activity, as response-specific processing in the monkey and human motor system starts from 150 ms after stimulus onset (Thorpe et al., 1996; Thut et al., 2000; Ledberg et al., 2007). In contrast, our results are well in line with previous studies describing similar unspecific responses characterized by very short latencies (<100 ms), as early as in the striate cortex, in both humans and monkeys (Foxe and Simpson, 2002; Ledberg et al., 2007). Unspecific early stimulus-evoked responses followed by more complex stimulus-specific processing, have been also described at the single neuronal level in the monkey dPMC and FEF during action selection tasks (Schall and Bichot, 1998; Cisek and Kalaska, 2005; see also Crammond and Kalaska, 1994). To date, the best explanation for these responses is that salient stimuli automatically attract motor attention before the preparation of the appropriate motor response, and suggests that a recruitment of the motor system by salient stimuli occurs well before its involvement in action planning. This early activation is posited to start an action specification and selection process in which action options are implemented as sensorimotor loops that are biased by several processes in, among others, prefrontal areas and sensorimotor loops (Cisek, 2007). This model predicts a phase in which action options are not yet specified or differentiated, but already present in the motor system. The early and undifferentiated response we report here fits this model.

The unspecific response was followed by a second peak preceding the movement onset. This activation was also found in most vPMC and mBA6 sites, but only in the dPMC this activity was modulated by the congruent, incongruent or neutral condition, showing a stronger response following the presentation of incongruent stimuli (see **Figure 2**, right panel). This data provide a mechanistic insight to the role played by the dPMC in the Eriksen flankers task, and in action selection more in general. It is known that flanking the central directional cue with incongruent directional stimuli elicits competing responses but, beside some indirect evidence on the involvement of the fronto-parietal circuits (Coulthard et al., 2008; Michelet et al., 2010; see also Cisek, 2008), little is known on the specific regions of the human brain in which this competition is solved. In contrast, electrophysiological studies on the monkey motor system provided mechanistic evidences to the role of the dPMC in action selection. Single unit recording during reach-selection tasks showed that, in the period of uncertainty between two opposite potential actions, the dPMC reflects both responses, while only after the information for selecting one action became available the representation of the chosen direction became strengthened (Cisek and Kalaska, 2005; see also Crammond and Kalaska, 1994; Gail et al., 2009). A similar multiple activation has been also shown in the adjacent FEF region during visual search tasks, showing an early modulation to all salient stimuli and a later one reflecting only the final selected target (Schall and Bichot, 1998). Similar mechanisms were also described in the posterior parietal cortex, in particular in the parietal reach region (PRR) connected with the dPMC, and in the lateral intraparietal sulcus (LIP) connected with the FEF (see Andersen and Cui, 2009 for a review). They may be objections that in these studies the monkeys were intensively trained to accomplish experimental tasks aimed to map perceptual decisions onto motor outputs and, as a consequence, their results could be affected by this intense training (see Kubanek and Kaplan, 2012). Our data shows that the findings in monkeys cannot be attributed to the intensive training in monkeys. Participants in our study only received a very short training session of less than two minutes. It remains to be seen whether these findings are induced by the forced-choice paradigm and can therefore be best understood as “task-set related” (Sakai, 2008), or whether the same principles underlie freely chosen actions as well.

According to the affordance competition hypotheses, whenever multiple activations, instructing opposite responses, appear simultaneously within a given region, they compete against each other for further processing (Cisek, 2007). The exact mechanisms for action selection, however, are still unknown. For example, the correct response could be enhanced until it crosses a threshold, or the wrong response could be suppressed until it drops below a certain threshold (or some delicate interplay between these two mechanisms). Although our study cannot provide concluding evidence, it does suggest that at least part of action selection is based on inhibiting the incorrect response.

Competition is assumed to be strongest upon incongruent trials, as concomitant rightward and leftward arrows automatically enhance the activity in favor of both left and right responses. Additionally, activity related to suppressing the wrong option is expected to be highest on the ipsilateral side, as this is the side that is not supposed to generate a response. **Figure 2**, left panel, clearly shows a significantly higher activation for incongruent trials at the ipsilateral side between 300 and 450 ms, being the phase right before the response.

In **Figure 2**, right panel, the same dataset from incongruent trials requiring a response with the hand ipsilateral to the recording site, is aligned to both the onset of the stimulus (upper part) and onset of the response (lower panel). The significant drop of activity before the movement onset, clear in the second alignment, suggests that the behavioral response depends on the suppression of the ipsilateral dPMC, and not on the increase of the contralateral dPMC (see left panel). So it seems that the competition between the two dPMC is won by the contralateral dPMC when the ipsilateral dPMC retreats. It is unlikely that this higher activation is caused by “general conflict,” rather than suppression, as conflict is expected to have an equal impact on both hemispheres (i.e., conflict is not related to the subsequent response), while our data shows greater activity only on the ipsilateral side. However, further experiments, including recordings in the right PMC are needed to substantiate this hypothesis.

We have shown that the dPMC shows a short latency activation, suggesting a relatively direct visual input. From monkey physiological findings it is known that area F2 – which is supposed to be the monkey counterpart of the caudal dPMC – receives visual input through MIP (medial bank of the intraparietal sulcus), and V6A (Matelli et al., 1998). The latter - which is suggested to have a human homolog (Pitzalis et al., 2013) - is of special interest here, as this parieto-occipital area connects to V2, V3, and V4, as well as V1 through V6 (Passarelli et al., 2011) and provides therefore relatively direct visual input. Moreover, about half of the neurons in V6A discharge in response to visual stimuli (Galletti et al., 1999), while the response in MIP seems to be stronger related to reaching movements.

Medial BA6 sites showed a significantly stronger activity during trials requiring a response with the ipsilateral hand after the first undifferentiated activation in dPMC, suggesting an involvement of this region in the inhibition of the wrong response (see **Figure 3**). This inhibitory role is in line with existing findings (Mars et al., 2009).

The vPMC shows only late onset activation that lasts well into the execution phase. However, since we have data of only few recording sites in vPMC, it is hard to draw firm conclusions from this.

If these findings are interpreted in terms of action selection through competition, it seems that the process of action specification start at the dPMC. The medial part of BA6 are subsequently involved in selecting the appropriate action, while vPMC is primarily involved in executing the action. In line with this model, previous intracranial recording from patients engaged in the Eriksen flankers task shows that incongruent trials correlate with a reduction of the theta phase alignment in the subthalamic nucleus (Zavala et al., 2013), that is indirectly connected with the caudal dPMC in the monkey (Saga et al., 2011). Together these findings suggest that the Erikson Flankers task evokes a response competition in humans similar to the ones found upon spatial color cues in monkeys. Of course, many other areas might be involved in the specification and selection processes, and based on this data set we cannot even conclude that these areas provide the backbone of the action specification and selection mechanism. More studies including more patients as well as more recording sites are needed.

While probably every empirical finding can be accommodated in both the classical and the embodied or enactivist approaches, we believe that these findings are much more in line with the latter frameworks. In principle, the classical framework would not predict an early and undifferentiated response in the motor cortices, as the motor system need not be active until a decision by cognitive processes have been made. That being said, it is possible to account for this early activation within this framework. One could for example argue that this is a non-functional side-effect of expecting *a* stimulus, while not knowing in advance *which* stimulus. Due to the instructions to respond as fast as possible, the motor system is brought to a heightened state of activation, and the mere appearance of the stimulus might be enough to cause a stir in the motor system. But it should be noted that this is a *post hoc* adjustment of the framework, and one would expect this to be a more general effect also present at vPMC and the mBA6. On the other hand, if action options are evaluated in the sensorimotor cortex, as suggested by the affordance competition hypothesis, the early activation is an essential component of the model (cf. also van Dijk et al.,’s 2008 “traffic regulator metaphor” for embodied embedded neuroscience). As soon as a stimulus appears, action options are activated as a sensorimotor loop. These loops are subsequently evaluated, biased, and selected predicting an early undifferentiated response, and a later differentiated response exactly as we reported. The fact that the dPMC shows two subsequent activations, one undifferentiated, one condition-dependent, strongly suggests a processing loop, rather than successive cognitive stages.

A possible objection to our interpretation of this result and the model of Cisek (2007) in enactivist terms (Varela et al., 1991; Hutto and Myin, 2013) is that merely replacing *one* representation of a to be planned action with *two* representations we still assume representationalism. However, the parallel action options should emphatically not be interpreted as representations, but rather as dynamic processes (Chemero, 2009). The options are not discrete states with fixed content, but develop over time in complex interaction with various brain structures as well as with the skeletomuscular system (Schurger and Uithol, in press). This dynamic nature cannot be accounted for using a representational approach. In all, the data discussed above suggest that the PMC accommodates or is part of a complex network in which action options are specified and selected in parallel. The nature and the pathways that favor one option over the other remains to be investigated, but our findings suggest that inhibition of the incorrect response, specifically by the medial part of BA6 and the dPMC plays an important role.

## Conflict of Interest Statement

The authors declare that the research was conducted in the absence of any commercial or financial relationships that could be construed as a potential conflict of interest.
